# Participant Experiences of an Infant Obesity Prevention Program Delivered via Telephone Calls or Text Messages

**DOI:** 10.3390/healthcare8010060

**Published:** 2020-03-16

**Authors:** Mahalakshmi Ekambareshwar, Sarah Taki, Seema Mihrshahi, Louise A. Baur, Chris Rissel, Li Ming Wen

**Affiliations:** 1 Faculty of Medicine and Health, Sydney School of Public Health, The University of Sydney, Camperdown, NSW 2006, Australia; 2 NHMRC Centre of Research Excellence in the Early Prevention of Obesity in Childhood, The University of Sydney, Camperdown 2006, Australia; 3 Prevention Research Collaboration, Charles Perkins Centre, Sydney School of Public Health, The University of Sydney, Camperdown 2006, Australia; 4 Health Promotion Unit, Sydney Local Health District, Camperdown 2050, Australia; 5 Sydney Medical School, The University of Sydney, Camperdown 2006, Australia; 6 New South Wales Office of Preventive Health, Ministry of Health, Liverpool 2170, Australia

**Keywords:** mHealth, infant obesity prevention, mother, process evaluation, mobile telephone calls, text messages, SMS, satisfaction, perception, childhood obesity

## Abstract

A 3-arm randomised controlled trial implemented in 2017, recruited participants from four Local Health Districts (LHDs) in New South Wales (NSW) to test an early obesity prevention program delivered via telephone calls (telephone) or text messages (SMS). This sub-study explored participants’ experience and satisfaction with the program. A multimethod design was used. Quantitative satisfaction questions were completed by participants when their child was six-months old. A purposive sample of participants with varying satisfaction levels was invited for in-depth qualitative interviews. Data were analysed using Excel (quantitative) and inductive thematic analysis (qualitative). Of the 1155 participants recruited: 947 (293 telephone; 338 SMS; 316 control) completed the six-month survey; 34 (14 telephone; 13 SMS; 7 control) were interviewed. Participants’ overall program satisfaction was 100% (telephone) and 85% (SMS). Participants’ qualitative responses demonstrated appreciation of: personalised stage-based information; opportunity to communicate with health professionals (telephone); linked Healthy Beginnings booklets and SMS mostly as nudges (SMS). There is a clear need for stage-based information, and supplemented modes of delivery i.e., text messages along with telephone calls; with text messages solely seen as nudges or reminders. However, individual preferences vary according to information needs at any given time, time constraints on new mothers and hence, multiple modes of information provision are recommended in order to reach a wider population and for better engagement. Choice and flexibility in mode of delivery has the potential to provide equitable access to information, empowering women with infants to practice recommended health behaviours for infant obesity prevention.

## 1. Introduction

Interventions for the prevention of obesity in early childhood have been delivered to parents, predominantly to mothers, mostly in trial settings. These interventions primarily focus on one or more behaviours such as breastfeeding, introduction of solids and physical activity. However, the mode of delivery of interventions differs between trials, for example: face-to-face via home visits [1,2]; face-to-face via parent groups in the community [3,4]; face-to-face via child care settings [5,6,7]; via internet [8]; via mobile applications or apps [9,10,11]; via text messages [12] and via telephone calls or/and text messages [13,14].

The Healthy Beginnings (HB) trial that delivered a program using a nurse-led home visiting model from the third trimester of pregnancy until two years of infant’s age was effective in the prevention of early childhood obesity, but home visits added significant costs (personnel, time and travel) [1,15]. Public health interventions delivered by telephone and internet-based approaches are proving to be cost-effective [16]. The increased use of, and reliability of mobile phones has led to a transformation in the way health interventions are delivered. Some examples of interventions delivered via mobile phones include: healthy lifestyle program for young adults [17]; healthy eating for children [18]; and health coaching for weight loss in adults [19]. The Communicating Healthy Beginnings Advice by Telephone Randomised Controlled Trial (CHAT RCT) has harnessed the increased use of mobile phone technology to communicate HB messages via telephone calls (telephone) or text messages (SMS) to pregnant women and women with babies to prevent early childhood obesity [13]. 

This multimethod study was conducted during the CHAT RCT intervention phase, to explore participant satisfaction, following the objectives of process evaluation frameworks [20,21]. Quantitative and qualitative approaches were used to understand participants’ actual experiences during the intervention phase, and to unpack the processes of implementation and behaviour change, which are important for future planning and scaling up to population-level. The aims of this paper were two-fold: (1) to explore participants’ experiences of participation in the program and perceptions of the utility, or otherwise, of the intervention contents delivered via telephone or SMS; and (2) to observe whether the perceptions and experiences differed between participants who received interventions via telephone or via SMS.

## 2. Methods

### 2.1. Study Context

The CHAT RCT was conducted across four Local Health Districts (LHDs) within New South Wales (NSW) Australia, where pregnant women were recruited at eight hospital sites between February and July 2017. The study protocol, eligibility criteria, recruitment process and outcomes are reported in detail elsewhere [13,22,23]. In brief, CHAT is a three-arm RCT that compares: mailed HB booklets plus telephone support (telephone); to mailed HB booklets plus text messages or Short Message Service (SMS); to the control arm. Interventions were stage-based and provided at six time points following key developmental milestones from the antenatal period (third trimester) until the end of first year of the infant’s life. The control arm participants were mailed general infant safety promotion materials. Usual care on infant development and safety is delivered by local child and family health services that are not mandatory.

### 2.2. Study Design

A multimethod sequential explanatory design with two phases was used to evaluate participants’ perceptions of the intervention contents and their experiences of participation in the CHAT RCT [24,25,26]. The design includes a quantitative phase and a follow-up qualitative phase. The purpose of this design was to use the qualitative results to further explain and interpret the findings from the quantitative phase [27]. 

#### 2.2.1. Phase 1—Quantitative Survey

Participant demographic characteristics on age, parity, country of birth, language spoken at home, household income, educational status, marital status and aboriginality were collected at baseline.

In the first phase, quantitative satisfaction data were collected from all CHAT RCT participants at the six-month follow-up survey. The survey included closed-item satisfaction questions about the program where participants rated satisfaction on a 5-point Likert scale (very satisfied to very unsatisfied) (Table 1). Questions also included whether they would recommend the program to other mothers and if they would like to participate in a further qualitative study. The survey was administered via telephone using computer assisted telephone interviewing (CATI). Participant responses were collected on a Microsoft Access database and exported to Excel for analysis.

#### 2.2.2. Phase 2—Qualitative Interviews 

In the second phase, qualitative interview data were collected post six-month survey (at around 12 months of infant’s age) and during the intervention phase. Participants who expressed interest in participating in further research at the six-month survey were eligible. Participants who met the inclusion criteria from the two intervention arms of the study and whose satisfaction levels with the program and program contents varied between satisfied and not satisfied were purposively selected by the first author (M.E.) to include a heterogenous sample [28]. Control participants were purposively selected to include participants who recommended or did not recommend the program to others. Participants were invited to participate in the qualitative interviews via email along with the information sheet and consent form. The email was followed by a text message sent to participants by a project team member for nomination of a suitable interview date and time. ME interviewed participants at the nominated dates and times. Prior to commencement of interviews, verbal consent was obtained and recorded.

Using the framework described by Patton [29], interviews were semi-structured to explore emerging themes associated with participants’ experiences and perceptions. An interview guide was developed following the satisfaction questions from the quantitative survey (Appendix B). Open-ended questions (tailored to the intervention arm of participants i.e., telephone, SMS or control) were asked, giving participants the opportunity to express their individual experiences. Prompts were used to elicit greater detail and for participants to elaborate on their experiences. Interview questions were pilot tested and modified for flow prior to administering to participants. All interviews were conducted by ME, who is trained and experienced in qualitative research methods. Interviews were conducted via telephone between March and June 2018 at a time convenient to participants (including evenings and weekends). This approach allowed flexibility in participation and was minimally intrusive [4,30,31,32]. The average interview duration was 20 min. Interviews were digitally audio recorded, transcribed verbatim and transcripts were cross-checked against recordings to ensure accuracy.

Authors M.E. and S.T. followed the principles of inductive thematic analysis [33] to generate an initial coding frame. Each researcher independently applied the coding frame to three interviews (one from each trial arm) and compared the analyses for consistency of codes, refined codes and eliminated discrepancies. The remaining transcripts were then coded by M.E. Following coding, themes were generated, refined in an iterative manner with four main themes identified: (1) overall opinion of the program; (2) mode of delivery; (3) intervention contents; and (4) contextual/psychosocial issues. 

### 2.3. Ethics Approval and Consent for Participation

The CHAT RCT is registered with the Australian Clinical Trial Registry (ACTRN12616001470482p) on 21 October 2016. This research was approved by the Sydney Local Health District Ethics Committee (approval number X16-0360 & LNR/16/RPAH/495). All interviewees provided verbal consent; permission to record the interview was also requested and obtained. Participants were offered a $20 gift voucher.

## 3. Results

Of the 1155 CHAT RCT participants, 947 (82%) completed the six-month survey. At the six-month survey, 761 (80%) participants agreed to participate in further research, of which 61 were approached for this qualitative study. Of the 61 participants who were approached, 36 (59%) participants consented to be interviewed of which two participants did not answer the telephone on three separate occasions. Qualitative interviews were conducted with 34 participants—14 (telephone), 13 (SMS) and 7 (control).

### 3.1. Demographics of Participants

Table 2 shows the demographic characteristic of the participants. The majority of participants were: first-time mothers; born overseas; spoke English at home; ≥30 years of age; university qualified; household income ≥AU$ 80,000; employed; and in a married or de-facto relationship (Table 2).

### 3.2. Participants’ Responses at the Six-Month Quantitative Survey

Table 3 reports the findings of participants’ responses, including all participants from the RCT and those who participated in this qualitative sub-study. 

In the telephone arm, the satisfaction levels of participants who were qualitatively interviewed were about the same as that of all telephone call participants. Participants who were qualitatively interviewed expressed 100 percent satisfaction with the ‘program’ and ‘type of advice provided by nurses’ (Table 3). 

In the SMS arm, the satisfaction levels of participants who were qualitatively interviewed were lower than that of all SMS participants except for satisfaction of ‘HB booklets’ where interviewed participants expressed 92% satisfaction (Table 3). 

Table 4 represents the satisfaction level of participants who were qualitatively interviewed. Most participants expressed satisfaction with the program and program contents.

### 3.3. Participants’ Satisfaction with Program at Qualitative Interviews

The four main themes are represented below: (1) overall opinion of the program; (2) mode of delivery; (3) intervention contents; and (4) contextual/psychosocial issues. Themes and sub-themes with illustrative quotes from the interviews are reported in detail in Appendix C.

#### 3.3.1. Theme 1: Overall Opinion of the Program 

Table 5 contains quotes representing participants’ overall opinion of program. Participants appreciated the stage-based delivery of the program received via telephone or SMS with linked booklets. The personalised messages provided comfort to those participants who considered themselves isolated when they were home with the newborn. Participants were concerned about the discrepancies on age of introduction of solids provided in the program compared to that received from other sources. First-time mothers rated the program highly and recommended the program to other first-time mothers. Second time mothers, newly arrived participants to the country and those without family support also felt that they benefitted from the program. Participants acknowledged the need for additional support and information as opposed to making an appointment to see a General Practitioner (GP).

#### 3.3.2. Theme 2: Mode of Delivery

Quotes related to participants’ perception on delivery of interventions via telephone calls or text messages or HB booklets are provided in Table 6.

##### Telephone Calls

Participants took comfort in receiving telephone calls from a nurse and appreciated talking to them about their concerns. The participants expressed their preference for receiving information that was tailored to their needs rather than given as a group. Despite reporting that the program was convenient, several participants found it difficult to make time for the calls. On occasions, it took several weeks for participants to be contacted by the intervention nurses. Participants were time poor due to responsibilities such as caring for infants. They appreciated and preferred the flexibility of communicating via telephone calls or text messages.

##### Text Messages

Participants found it convenient to read the text messages in their own time when baby was asleep. Messages were reassuring and gave participants the confidence to continue breastfeeding. Other participants considered text messages as mere reminders or nudges that did not provide additional benefits to them in comparison to the booklets. Participants expressed a need for choice of receiving interventions and recommended combination modes of text messages plus telephone calls or text messages plus email.

##### HB Booklets

Participants considered HB booklets as handy resources that they could refer to anytime and a resource they could share with their partners. HB booklets were also used to present credible information to their family and was used as a source of authority to encourage practice of desired behaviours. Participants from culturally and linguistically diverse background found the booklets easy to understand. These participants preferred the booklets in comparison to conversing in English, which they found difficult at times.

#### 3.3.3. Theme 3: Intervention Contents

Participants provided their opinion on contents of the stage-based intervention, key quotes are included in Table 7.

##### Breastfeeding

Generally, participants without breastfeeding issues considered the advice provided on breastfeeding was useful. Participants who experienced issues such as low milk supply, mastitis and latching were likely to explore services beyond the program to receive help in managing those issues. Participants who were unable to continue breastfeeding for as long as specified, felt like they were judged by the program providers.

##### Introduction to Solids

Although most participants found the information on solids useful, there was a general lack of awareness on when to start solids. Participants reported that they sought information on solid feeding from other sources including family members, doctors, the internet and classes. In some instances, it appeared that family members had a strong influence on participants’ decisions with regards to feeding solids, while for others, the program enabled participants to convince family members on choice of food for infant. In particular, the telephone calls with the nurses were likely to influence participants’ decision to delay the introduction of solids until after 6 months. Some mothers made an effort to feed according to the guidelines for their current child even if they did not for their previous child.

##### Tummy Time 

Participants needed to be reminded, and appreciated the reminders, that encouraged them about early commencement of practice of tummy time. Some participants expressed they were too busy for tummy time. 

##### Screen Time

Telephone calls and text message reminders on tummy time and screen time recommendations raised participants’ awareness. Some participants allowed infants to watch television in order for them to carry out household chores or to go to the toilet. 

##### Sleep and Settling

Some participants acknowledged that strategies and support provided via telephone to manage infant’s sleep were helpful. Other participants expressed that they struggled to get information on how to settle their babies to sleep and reported seeking support beyond the program to help manage their infant’s sleeping pattern.

##### Goal-Setting

Although participants considered goal-setting important, they experienced challenges with achieving goals due to balancing parenting responsibilities, doing household chores or returning to work. Participants appreciated the follow-up emails sent by intervention nurses that participants shared with family members. Goal-setting strategies also helped participants manage baby’s reflux, breastfeeding issues or preparation of home-made infant solid foods.

#### 3.3.4. Theme 4: Contextual/Psychosocial Issues

Difficulties were expressed by participants during participation in the trial (Table 8). Participants were anxious on how to adjust to becoming a new mother and expressed the need for more information, particularly in the early stages after childbirth. A few participants reported feeling overwhelmed by the advice and felt that there was a high expectation of mothers. Participants who had infants with developmental issues were not able to follow the stage-based advice since they needed to deal with the situation at hand.

#### 3.3.5. Control Arm Participants’ Perception

Participants in the control arm considered that the information in the control booklets was very general in nature and expressed a need for support and information more broadly and particularly on feeding of solids. This program did not meet their needs and they sought help from external sources. Despite being in the control arm, participants were satisfied to remain in the program.

“When I really needed help with breastfeeding or questions about solids or brushing teeth or developmental milestones, I would need to either make an appointment with the early childhood health centre or call ABA (Australian Breastfeeding Association) or Tresillian (an early parenting service) or try to find a doctor.” Control participant C4.

## 4. Discussion

We conducted a process evaluation of the intervention phase of the CHAT RCT to understand participants’ actual experiences of the program; and observed any differences between the perceptions of telephone and SMS participants. Almost all participants in the telephone arm and several participants in the SMS arm reported participation in the program as positive, valued the stage-based information, appreciated the flexibility of intervention provision particularly in relation to the mode of intervention delivery via telephone calls or text messages with linked HB booklets. Based on the *p*-values in Table 2, it can be interpreted that the interviewed participants were representative of the CHAT population. Many participants appreciated the information in the HB booklets and considered them as handy resources and referred to them at their convenience. In particular, first-time mothers found the program very useful and recommended that the program be delivered to all first-time mothers. Participants’ responses and experiences demonstrate the need for information provision and support. However, due to variable information needs and participants’ time constraints, their preference is for flexibility and choice in mode of delivery.

Participants in the telephone arm valued the telephone calls since they considered the calls were individually tailored to their needs. Participants attributed positive perceptions of the program to the opportunity for them to ask questions of nurses at the time of intervention delivery that enabled discussion of issues and in some instances, participants expressed that the nurses helped them resolve issues and alleviated the need to visit health professionals. Text messages were perceived as convenient by participants in the SMS arm since they could read the messages in their own time, however, the majority of participants preferred the HB booklets to text messages. Many considered the text messages as mere nudges or reminders supplementary to the HB booklets. A few in the SMS arm stated that the text messages lacked personalisation. Participants in the control arm also expressed the need for support and information but were satisfied to remain in the program despite receiving information of a general nature only.

The vast majority of participants welcomed the stage-based interventions delivered to them via telephone calls or text messages with the linked booklets and this has been shown in earlier home-based studies [1]. Although home-based interventions were successful in reaching women, they were resource, time intensive with several logistical issues involved in home visiting. In this program, the convenience of receiving stage-based advice and support via telephone calls or text messages and when participants needed them were appreciated by majority of participants. Studies that reported participants’ views on delivering adult and infant obesity prevention interventions via telephone calls, text messages or apps generally report high satisfaction rating and have been regarded favorably by participants [9,11,12,16,17,34].

The finding that some participants considered it difficult to adhere to the advice provided, and they considered this program burdensome on top of other commitments and responsibilities, has been shown in other studies [3,35,36]. Lack of compliance with the program was encountered in other infant obesity prevention programs delivered via more traditional modes e.g., face-to-face, and attributed to time constraints, travel distances, busy schedules and return of participants back to work [35,36,37]. Programs need to acknowledge competing commitments of participants and consider providing flexibility and choice. Participants expressed a need to have choice of delivery modes, for example, telephone calls or text messages or combination of both. Their views are similar to those expressed by participants in other obesity prevention programs that suggest innovative approaches to delivering interventions [35], where text messages were considered as supplementary to telephone coaching calls [12,17,34], or where participants preferred a combination of telephone calls and/or emails with social media or online platforms due to changing needs and to enhance engagement [8,9,10,38].

One important finding of this study was related to breastfeeding and solid feeding advice where advice is sought by mothers. For example, credible, non-judgmental support for breastfeeding and infant solid feeding provided by health professionals was appreciated and welcomed by participants. Participants without major breastfeeding issues considered the program helpful but those who faced breastfeeding issues expressed that they needed hands-on, face-to-face practical support. Credible content from reliable sources were valued highly by participants in other infant obesity prevention programs and contributed to increased engagement [9,11]. A few participants in this program expressed concerns that the information they received on timing of introduction to solids conflicted with the information they received from external sources.

Many participants in this program used screen time for infants as a coping strategy in order to undertake household chores, which is consistent with many other studies, and at times was viewed as an educational tool [6,7]. Our results reflect that sleep and settling are major issues that affect mothers and their children. Although studies have provided interventions for sleep and settling prior to this study [39,40,41,42,43,44], perceptions of participants have not been explored previously. In this study, several participants said that they struggled with their infant’s sleep and settling and preferred to receive more information on sleep and settling. Goal-setting was appreciated by some participants but most participants commented that they did not have the time to adhere to interventions on self-care through exercise. While constructive goal setting was viewed positively [34], the capacity of physical activity interventions for parent behaviour modification is limited [16].

### Strengths and Limitations

A key strength of this research was the use of multimethod sequential explanatory research design, where qualitative results were used to further explain and interpret the findings from the quantitative phase. The quantitative and qualitative phases were conducted with participants at a time close to the intervention, for completeness of the recollections retrieved by participants regarding their experiences [45,46]. Additionally, converging evidence through triangulation of the quantitative and qualitative data adds to the strength of this study [24,25,26,27].

Some participants were challenged with health/psychosocial issues post-childbirth that prevented them from active participation. There was an option for participants to withdraw at any time but there were no other mechanisms in place for the team to monitor participants’ changing needs, especially of those in the SMS arm. In an RCT environment, there was limited to no flexibility for variation to accommodate the changing needs of mothers. The program was offered to participants fluent in English in an urban setting, therefore the results may not necessarily reflect the perceptions of women who were Indigenous, or from culturally and linguistically diverse backgrounds or remote or rural areas. This is important as the program has the potential to support families in isolated, rural and remote locations and further work would need to be carried out before implementation in this population. 

## 5. Conclusions

Process evaluation of the intervention phase of the CHAT RCT improved our understanding of participants’ perceptions of and satisfaction with the program. Participants’ responses are indicative of their appreciation of the program since it met their needs, particularly information relating to breastfeeding, solid feeding and infants’ sleep. Delivering health promotion messages via telephone calls or text messages has the potential to provide equitable access to information by women from various socio-economic and culturally diverse backgrounds. These findings demonstrated that information-provision can potentially increase women’s health literacy skills and empower them to practice recommended health behaviours for infant obesity prevention.

Participants’ responses indicate a clear need for stage-based information provision with preference of choice and flexibility in mode of intervention delivery due to their changing needs. Future translation or scaling-up of the program should explore the possibility of integrating this program with existing programs such as the New South Wales Get Healthy and the Get Healthy in Pregnancy Services; with appropriate referral pathways to address participants’ psychosocial needs during and post pregnancy. There is a clear need for supplemented modes of delivery i.e., text messages along with telephone calls; with text messages solely seen as nudges or reminders. However, individual preferences vary according to information needs at any given time, time constraints on new mothers and hence, information provision via multiple modes is recommended in order to reach a wider population and for better engagement.

## Figures and Tables

**Table 1:** Satisfaction questions administered at the six-month quantitative survey.

Satisfaction Questions	Telephone Participants	SMS Participants	Control Participants
Overall Healthy Beginnings program	✓	✓	
Healthy Beginnings booklets	✓	✓	
Receiving advice on baby’s growth and your health through telephone	✓		
Receiving advice on baby’s growth and your health through SMS		✓	
Timeliness of calls from nurses	✓		
Timeliness of the SMS		✓	
Type of advice nurses provide you with	✓		
Type of advice the SMS provide you with		✓	
Quality of service provided	✓	✓	
Responses you have received after sending an SMS		✓	
Would you recommend the Healthy Beginnings program to other mothers?	✓	✓	✓

**Table 2:** Demographic characteristics of participants.

Characteristic	Category	All CHAT RCT Participants *N* = 947	Non-Interviewed Participants *N* = 913	Interviewed Participants *N* = 34	*p*-Value *
		*n* (%)	*n* (%)	*n* (%)	
Intervention arm	Telephone calls + Booklets	293 (31)	279 (30)	14 (41)	0.2249
	Text messages + Booklets	338 (36)	325 (36)	13 (38)	
	Control (usual care)	316 (33)	309 (34)	7 (21)	
Parity	First-time mother	515 (54)	492 (54)	23 (68)	0.1138
	Not first-time mother	432 (46)	421 (46)	11 (32)	
Country of Birth	Australia	361 (38)	344 (38)	17 (50)	0.1464
	Other	586 (62)	569 (62)	17 (50)	
Aboriginal status	Non-Aboriginal	925 (98)	892 (98)	33 (97)	0.8077
	Aboriginal	22 (2)	21 (2)	1 (3)	
Language spoken at home	English	525 (55)	502 (55)	23 (68)	0.1446
	Other	422 (45)	411 (45)	11 (32)	
Age (years)	≥30	661 (70)	633 (69)	28 (82)	0.1044
	<30	286 (30)	280 (31)	6 (18)	
Education	University	642 (68)	615 (67)	27 (79)	0.1398
	Other	305 (32)	298 (33)	7 (21)	
Household income	≥AUS$80,000	551 (58)	525 (58)	26 (76)	0.0885
	<AUS$80,000	301 (32)	295 (32)	6 (18)	
	Don’t know	95 (10)	93 (10)	2 (6)	
Employment status	Employed	604 (64)	577 (63)	27 (79)	0.0534
	Other	343 (36)	336 (37)	7 (21)	
Marital status	Married/de-facto partner	887 (94)	855 (94)	32 (94)	0.9192
	Other	60 (6)	58 (6)	2 (6)	

* Chi-square test of independence was performed to examine the relation between characteristics of interviewed and non-interviewed participants.

**Table 3:** Aggregate participants’ responses to satisfaction questions at the six-month survey.

Satisfaction Questions	Telephone Participants		SMS Participants		Control Participants	
	All Participants (*N* = 293)	Participants Interviewed (*N* = 14)	All Participants (*N* = 338)	Participants Interviewed (*N* = 13)	All Participants (*N* = 316)	Participants Interviewed (*N* = 7)
	Very Satisfied/ Satisfied *n* (%)	Very Satisfied/ Satisfied *n* (%)	Very Satisfied/ Satisfied *n* (%)	Very Satisfied/ Satisfied *n* (%)	Very Satisfied/ Satisfied *n* (%)	Very Satisfied/ Satisfied *n* (%)
Overall Healthy Beginnings programme	286 (98)	14 (100)	294 (94)	11 (85)		
Healthy Beginnings booklets	278 (95)	13 (93)	293 (94)	12 (92)		
Receiving advice on baby’s growth and your health through telephone	284 (97)	13 (93)				
Receiving advice on baby’s growth and your health through SMS			283 (90)	9 (69)		
Timeliness of calls from nurses	275 (94)	12 (86)				
Timeliness of the SMS			282 (90)	8 (62)		
Type of advice nurses provide you with	286 (98)	14 (100)				
Type of advice the SMS provide you with			275 (88)	9 (69)		
Quality of service provided	288 (98)	12 (86)	299 (96)	11 (85)		
Responses you have received after sending an SMS			212 (68)	5 (38)		
Would you recommend the Healthy Beginnings program to other mothers?	290 (99)	13 (93)	330 (98)	11 (85)	298 (94%)	5 (71%)

**Table 4:** Satisfaction of participants (who were interviewed) with program and program contents at the six-month survey.

Satisfaction Questions	Telephone Participants Interviewed (*N* = 14)	SMS Participants Interviewed (*N* = 13)
	Very Satisfied/ Satisfied *n* (%)	Very Satisfied/ Satisfied *n* (%)
Overall Healthy Beginnings programme	14 (100)	11 (85)
Healthy Beginnings booklets	13 (93)	12 (92)
Receiving advice on baby’s growth and your health through telephone	13 (93)	
Receiving advice on baby’s growth and your health through SMS		8 (62)
Timeliness of calls from nurses	12 (86)	
Timeliness of the SMS		8 (62)
Type of advice nurses provide you with	14 (100)	
Type of advice the SMS provide you with		9 (69)
Quality of service provided	12 (86)	11 (85)
Responses you have received after sending an SMS		5 (38)

**Table 5:** Quotes supporting participants’ overall opinion of the program.

Overall Opinion of the Program
“The booklet sort of explains what to start feeding them, when the time is right and … just new things that they are going to be learning at that stage …” (Satisfied telephone participant T10)
“When I receive the text messages, when alone at home with the baby, when you get a message like that, it sort of brightens your day … oh, someone is thinking about me …” (Satisfied SMS participant S3)
“I also found it useful just to engage with somebody else … sometimes you become isolated. It’s good to have somebody else check in on you now and then … I could actually discuss topics that reflected my need.” (Satisfied telephone participant T2)
“I have to say for that bit, I was a little bit confused because there was so much conflicting advice … I felt like there was a lot of conflicting advice out there about when I should have started her on solids.” (Unsatisfied SMS participant S4)
“I would highly recommend other mothers to be a part of this program. It has been useful and it has been a really great way of adjusting to being a new mother.” (Satisfied SMS participant S1)
“I would like every, at least first-time mum, to experience all this. I think it’s a blessing to have such a program.” (Satisfied telephone participant T12)
“I’m a second time mother. I forget from the first one … The booklet is new information for me. May be the first one I do the wrong thing, you know … so this one is good for me.” (Satisfied telephone participant T5)
“Some mothers do want some extra support and going to a GP is hard and you need to make an appointment, so this is over the telephone, it’s easier to access information.” (Partially satisfied text message participant S13)

**Table 6:** Quotes related to participants’ perception of mode of delivery.

Mode of Delivery
Telephone calls
“It was good to talk to a nurse … it was comforting to know it had come from a health professional rather than me having to go down to the doctor and probably get the same answer.” (Satisfied telephone participant T13)
“The phone calls have been beneficial … individualised feedback, they would explain things until I understood them myself and not just as if it was to a whole group of people.” (Partially satisfied telephone participant T3)
“It was always very difficult coordinating the phone call time … Maybe because I don’t have a routine baby. I wouldn’t know for sure when I was going to be free on any day, so often I would call when it was a good time for me, but of course the nurses would be busy. Often we’d play phone tag for weeks trying to get hold.” (Satisfied telephone participant T1)
“It was good to talk to a nurse … if you had concerns about something … I got to ask them a question via text message and they came back to me the next day with a response.” (Satisfied telephone participant T13)
Text messages
“I would be thinking about something was going on with breastfeeding or something and the next day I would get, just by chance a message … it kind of made me go, yes I am doing the right thing … I think I was probably happy with the text messages because I often didn’t have time for a telephone call.” (Satisfied SMS participant S5)
“I didn’t really benefit very much from the text messages. I did look at the text messages … just found the booklets more useful … I feel like it was a nice addition, but I don’t feel like it’s necessary to get the text messages.” (Satisfied SMS participant S1)
“But maybe a mix of the two…because text messages you are not as likely to remember it all, so may be a phone call or something might have built that… I think that everyone is different, so I think you need to give kind of a choice…probably recommend a couple of phone calls in there.” (Satisfied SMS participant S5)
HB booklets
“It was all useful to me…my husband, he also found it really useful, so we both kind of have the same views on the content.” (Satisfied SMS participant S1)
“It was handy to have a source of authority to say that this is why I’m not feeding him those things.” (Satisfied telephone participant T1)
“It was described in a very easy way, the booklet. I can understand… so it was easy. In terms of the nurses’ calls may be sometimes a little bit of tension but it’s fine.” (Satisfied telephone participant T7)

**Table 7:** Participants’ quotes on intervention contents.

Intervention Contents
Breastfeeding
“I saw a lactation consultant and I also went to the breastfeeding drop-in clinic. I also went to a twins breastfeeding class at [name of hospital].” (Partially satisfied SMS participant S2)
“I stopped breastfeeding at five months old … we had breastfeeding issues … so I kind of felt like, if I didn’t breastfeed her that I was being judged.” (Satisfied SMS participant S1)
Introduction to solids
“Information on introduction to solids was useful because I have no idea what to give the baby when he can start solids … In terms of feeding solids may be some more information is better because … I am still feeding him by the spoon.” Satisfied telephone participant T4
“Well, my husband’s family were pushing very strongly that I feed him things like custard, sweetened with honey and things like that … It was handy to have a source of authority.” (Satisfied telephone participant T1)
“The nurse said don’t give solids to the baby at three months … The reason why nurse said not to bring in solids is because his digestive system was just growing.” (Satisfied telephone participant T12)
“The only thing that really stood out was the breastfeeding fine until six months and start solids. I didn’t do that with my other children. I only done it with this one because I read it in the book. I done it a lot earlier.” (Satisfied SMS participant S10)
Tummy time
“Sometimes mothers know a lot of things but when someone talks to them about tummy time … it’s kind of like a reminder … oh no, that’s right, we’ve got to do that today.” (Satisfied telephone participant T6)
“The booklets reminded me about tummy time and I really got onto tummy time …” (Partially satisfied telephone participant T14)
“I don’t do it a lot actually, just because I was so busy.” (Partially satisfied SMS participant S2)
Screen time
“A lot was stressed on tummy time and about screen time, and I’m glad I wasn’t doing screens anyway …” (Satisfied telephone participant T6)
“My baby watches TV. I know she shouldn’t … I need to do things like go to the toilet, or cook dinner … it’s just in the background.” (Satisfied SMS participant S1)
Sleep and settling
“The nurses used to tell me how many hours our baby should sleep and how to put the music on or try to give them the environment where he can sleep more without disturbing.” (Satisfied telephone participant T12)
“I definitely supplemented especially the sleep parts with some other books that I had… that was the one thing she struggled with unfortunately … Tresillian (an early parenting service) even came out to help us as well … I think I needed a bit more information on that.” (Unsatisfied SMS participant S4)
Goal-setting
“I liked how they had goals … they summarised the call and sent you emails with your goals written down. Sometimes I would pass those emails on to my family just to let them know what stage I was up to …” (Satisfied telephone participant T1)

**Table 8:** Quotes underpinning participants’ contextual/psychosocial issues.

Contextual/Psychosocial Issues
“I probably struggled initially to adjust to the life change … maybe if there was more information at the beginning that was around … a lot of women feel the same way … adjusting to being a mother is really hard …” (Satisfied SMS participant S1)
“I guess the one thing that I probably struggled with was there’s a lot of expectations on mothers … the advice that you get in terms of … it’s incessant.” (Satisfied telephone participant T2)
“I found all of that kind of information (feeding and eating) frustrating for me personally because I wasn’t able to follow it due to swallowing difficulties … I’d feel like my kid was really delayed because he couldn’t do the things … in the literature or he couldn’t do the things other kids were doing” (Satisfied telephone participant T10)

## Abbreviations

CHAT: communicating healthy beginnings advice by telephone; HB: healthy beginnings; LHD: local health district; NSW: New South Wales; RCT: randomised controlled trial; SMS: Short message service.

## Researcher Description

All researchers of this research study were also part of a larger study evaluating the effectiveness of the CHAT RCT. Authors M.E., S.T., S.M., L.A.B., C.R., L.M.W. are public health researchers at various professional stages from early to senior career, experienced with evaluating early childhood obesity prevention interventions in populations. The researchers bring to this study expertise in quantitative and qualitative research skills, as well as diverse multi-disciplinary backgrounds in medicine, dietetics and science. To ensure the research team remained impartial, L.A.B. as the director of EPOCH CRE was not involved in the data collection or analysis. This study was conducted as part of M.E.’s doctoral research of process evaluation of the CHAT RCT. Detailed quality assessment against CoreQ in Appendix A.

## Appendix A. Quality Assessment against Consolidated Criteria for Reporting Qualitative Studies (COREQ)

**No.**	**Item**	**Question**	**Description**
Domain 1: Research Team and Reflexivity Personal Characteristics			
1.	Interviewer	Which author(s) conducted the interviews?	First author (ME) conducted interviews in Sydney.
2.	Credentials	What were the researchers’ credentials?	ME: MHS (Hons) ST: PhD SM: PhD LAB: MBBS, PhD CR: PhD LMW: PhD
3.	Occupation	What was their occupation at the time of the study?	ME: Doctoral candidate ST: Postdoctoral Fellow SM: Senior Research Fellow LAB: Professor CR: Director LMW: Manager
4.	Gender	Was the researcher male or female?	ME: Female ST: Female SM: Female LAB: Female CR: Male LMW: Male
5.	Experience and training	What experience or training did the researcher have?	ME: Formal training in qualitative methods; completed graduate-level coursework in qualitative research ST: Completed thesis studies on qualitative inquiry SM: Conducts original research in qualitative inquiry LAB: Clinical researcher with some experience in qualitative inquiry CR: Leads several qualitative research studies LMW: Leads several qualitative research studies.
Relationship with Participants			
6.	Relationship established	Was a relationship established prior to study commencement?	Interviews were conducted via telephone and there were no pre-existing relationships between participants and the interviewer.
7.	Participant knowledge of the interviewer	What did the participants know about the researcher?	Participants were not given information about the interviewer beyond a brief introduction in an invitation email and in the participant information sheet provided with the email that described the interviewer’s role in this sub-study.
8.	Interviewer characteristics	What characteristics were reported about the interviewer?	None.
Domain 2: Study Design Theoretical Framework			
9.	Methodological orientation	What methodological orientation was stated to underpin the study?	Qualitative description.
Participant Selection			
10.	Sampling	How were participants selected?	Purposive sampling.
11.	Method of approach	How were participants approached?	By email and text messages.
12.	Sample size	How many participants were in the study?	34.
13.	Non-participation	How many people refused to participate or dropped out? Reasons?	Sixty-one women were approached via email and 25 participants passively rejected our invitation to participate without providing reasons other than general lack of interest in the study. 2 women who consented to participate did not answer the telephone on three separate occasions. 34 participants were interviewed.
Setting			
14.	Setting of data collection	Where was the data collected?	Participants’ choice of location since they were contacted via mobile telephones.
15.	Presence of non-participants	Was anyone else present besides the participants and researchers?	None at the researcher’s end, unsure of who else was present at the participants’ end.
16.	Description of sample	What are the important characteristics of the sample?	Women who participated in the CHAT RCT trial and who agreed to participate in further sub-studies. Women caring for infants who were around 12 months of age with varying satisfaction levels at the six-month quantitative survey.
Data Collection			
17.	Interview guide	Were questions, prompts, guides provided by the authors? Was it pilot tested?	Yes. The guide was improved/refined throughout pilot testing and during data collection process.
18.	Repeat interviews	Were repeat interviews carried out?	No.
19.	Audio/visual recording	Did the researcher use audio or visual recording to collect the data?	Interviews were audio-recorded.
20.	Field notes	Were field notes made during and/or after the interviews?	Yes. Field notes were made during and immediately following interviews.
21.	Duration	What was the duration of the interviews?	Approximately 20 min.
22.	Data saturation	Was data saturation discussed?	Yes.
23.	Transcripts returned	Were transcripts returned to participants for comment or correction?	No.
Domain 3: Analysis and Findings Data Analysis			
24.	Number of data coders	How many coders coded the data?	Two researchers (ME and ST) coded one set of data independently. Codes and categories were discussed and refined within the research team. Following this, ME coded the remaining data
25.	Description of the coding tree	Did authors provide a description of the coding tree?	Yes.
26.	Derivation of themes	Were themes identified in advance or derived from the data?	Derived from the data.
27.	Software	What software, if applicable, was used to manage the data?	Microsoft Word.
28.	Participant checking	Did participants provide feedback on the findings?	No.
Reporting			
29.	Quotations presented	Were participant quotations presented to illustrate the themes/findings? Was each quotation identified?	Yes, some quotations were included within the manuscript and all quotations in a separate table. Quotations were identified by category.
30.	Data and findings consistent	Was there consistency between the data presented and the findings?	Yes.
31.	Clarity of major themes	Were major themes clearly presented in the findings?	Yes.
32.	Clarity of minor themes	Is there a description of diverse cases or discussion of minor themes?	Both major and minor themes were discussed.

## Appendix B. Qualitative Interview Guide

**Communicating Healthy Beginnings Advice by Telephone (CHAT): a three arm RCT**

**A qualitative study to explore participants’ experience and satisfaction with the CHAT Trial**

Participant semi-structured interviews regarding experience and satisfaction with the CHAT Trial

**Things to note before interview takes place**:

- That verbal consent has been obtained.
- Acknowledge that you are a researcher conducting this interview for the participant experience of the Healthy Beginnings messages and the CHAT Trial.
- Explain that the interview is recorded for research purposes. In the recording state ‘for recording purposes please confirm that you consent to being recorded for this interview’.

**Interview begins**

*Now I will commence my interview with a general question*

1. Leading question/s
  1. How has your experience of becoming a mother been? (modify it to multiparous women—How has your experience of becoming a mother been this time around?)
  2. What motivated you to participate in the Communicating Healthy Beginnings Advice by Telephone (CHAT) program? What were your reasons for participation in the CHAT program?
2. It is the intent of the following questions to be as open-ended as possible in order to gather rich data on participant experience of the CHAT program

*I will now ask questions about your experience and satisfaction with the CHAT program*

3. Participant Perception
  3. I would like you to tell me about your experience of participating in the CHAT Trial? What is it like to be part of the CHAT program? For instance, what was your involvement when you received (a telephone call from the Healthy Beginnings (HB) Nurse) or (a text message from the HB team) or (resources from the HB team)?
  4. Has the CHAT program met your expectations so far? How have they met or not met your expectations?
    Prompts
    (a) How much did you use the resources/advice provided by CHAT trial team?
    (b) Was there anything about the CHAT program that you particularly liked?
    (c) Was there anything that you particularly disliked?

Participant Perception of mode of delivery of program

**Telephone**

As part of the CHAT program you would have received telephone calls from our nurses at different stages of your baby’s growth to provide you telephone support with looking after your baby including feeding, activity and sleep patterns. You were also given advice to look after your health.

5 What has been your experience of the telephone calls with the nurses?
  Prompts
  Have the calls been useful?
  Have the calls met your needs?
  (a) What is your impression on the number of calls that were made throughout the program until now?

**Text message**

As part of the CHAT program you were sent Healthy Beginnings text messages at different stages of your baby’s growth to provide you support with looking after your baby including feeding, activity and sleep patterns. You were also sent messages to look after your health.

6 Do you remember receiving Healthy Beginnings text messages?
  IF YES
  (a) What has been your experience with the text messages?
    Prompts
    Have the messages been useful?
    Have the messages met your needs?
  (b) What is your impression on the number of text messages that were sent throughout the program until now?
  If NO
  (c) If you haven’t read the messages, did you ignore all the messages?
  (d) What would make you open the text messages?
    Prompt
    Would you have preferred other contents, if so what?]
    Would you have preferred it be sent a different time or stage of your baby’s growth?

**Participant perception of intervention booklets**

As part of the CHAT program you would have received 5 Healthy Beginnings booklets (3rd trimester, 0–2, 2–4, 4–6, 6–8)

7 Do you remember receiving the booklets?
8 If yes
  (a) How useful did you find the information in the booklets (prompt: easy to understand, too little/much)?
  (b) Were there any times you struggled to find the information you wanted?
  (c) Did the booklets meet your information needs for feeding and raising baby or did you refer to other sources of information?
  (d) What did you think of the look of the booklets (Prompt: colours, font, images)?

Intervention acceptance

*Next, I will ask about your experience with the interventions that were delivered so far*

9 The Healthy Beginnings booklets had information to support you with feeding your baby including breastfeeding, age to introduce solids to your baby, the type of foods to introduce:
  (a) In what way if any did this information influence the way you fed your baby?
  (b) Before looking at the Healthy Beginnings booklets, did you know much about:
    - Breastfeeding
    - Age to introduce solids to your baby
    - The type of foods to introduce to your baby
10 The booklets also covered information about active play for your baby including tummy time and activities to do with your baby
  (a) In what way if any did this information influence the activities you did with your baby?
  (b) Before looking at the Healthy Beginnings booklets, did you know much about:
    - Tummy time
    - Sleep and settling
11 In the CHAT Trial, you were given some key messages at different stages of your baby’s growth. Were they delivered at an appropriate time of your baby’s growth and were they easy to follow?
12 Do you recall the Healthy Beginnings (HB) messages such as “Breast is best”, “No solids for me until 6 months”, “Tummy time is fun”?
  Did these messages influence you in practicing these behaviours more / less or about the same?
13 Have you changed any of the practices / behaviours as a result of the HB advice / message you received? If you have changed practices / behaviours which practices have you changed? And what made you adapt the new behaviour / practice?

Emerging issues

*I will now ask you a few more questions to identify any other issues that you experienced as a participant of the CHAT Trial*

14 What were some of your goals or aims? How much was your goal selection influenced by text messages or telephone call from a nurse or visit to a baby health clinic?
15 Did you experience difficulties in using the CHAT program or adopting the messages delivered? What were they?
16 Are there any other issues/ concerns for mothers like yourself that need to be addressed, that was not addressed in the CHAT program?
17 Do you think mothers like you will benefit from the CHAT program? Why or why not?
18 If you were not part of CHAT program, where would you have obtained the support or information you would have needed?

Context

19 We understand that you have a busy lifestyle, were there any competing issues in life that enabled you to participate actively or prevented you from participating actively in the CHAT program?

Prompts

Work commitments, family practices, cultural background

Encouragement of family members, discouragement of family members

Conclusion

This ends the formal questions for today. Are there any other issues about the program that you would like to add or comment on? Thank you for your time.

## Appendix C. Quotes from Interviews with Participants Illustrated by Themes and Sub-Themes

**Theme**	**Sub-Theme**	**Participant Quotes**
1. Overall opinion of program	1.1 Reasons for participation	I thought that it might help people. It’s a public health initiative and it’s a really serious issue, childhood obesity. It sets people up for a lifetime of chronic health conditions if they’re obese as babies and toddlers. Telephone call participant T1 Someone came to me, at the hospital, during one of my visits. They gave me a bit of background on the study. They sent up leaflets and pamphlets at a timely period for when having a baby … Being a first-time mom, I had no idea what I was doing. So, it was able to kind of tell me what to expect, which was great. Text message participant S1 Yeah, even if it’s with just the general, I was just getting a general level of information. I do feel like it made me feel a bit, well, connected … I think having an option for the phone contact or text message I think would probably be good for a lot of women. Because there are times that I get out, if they’re not going to leave the house to go to a mother’s group or community care then I think it’s a really good goal to have one person that can assist you with that sort of stuff. Control participant C1 I do not have my mother here … so I thought that they are experienced people, so they would definitely help me in looking after my baby … that is the thing which motivated me to … participate. Control Participant C3
	1.2 Information and support	That’s a really tricky thing in terms of the reliability of anything. Often, even from government publications, you can be getting conflicting messages and answers. Telephone call participant T1 After the support’s dried up and after your partner’s back at work, and how things are going later on. So many women I know have quite a really difficult first year. Yeah, I think the telephone consultations would be very useful if everyone had access to them ideally. Telephone call participant T1 If it is a government or a website of a government program, then you … know it is credible. Sources that don’t have that sort of backing like mother’s groups forums … just seeing what other people’s experiences are … and you know you are not the only one experiencing certain things. Telephone call participant T8 Some mothers do want some extra support and going to a GP is hard and you need to make an appointment, so this is over the telephone, it’s easier to access information. Text message participant S13 When I really needed help with breastfeeding or questions about solids or brushing teeth or developmental milestones I would need to either make an appointment with the early childhood health centre or call ABA or Tresilian or try to find a doctor. Control participant C4
	1.3 Feedback	It was like somebody holding your hand along the way. You knew that somebody knows that you are out there, that they know your baby’s name…rather than having to reach out for resources or information specific to you which you may not have time for or motivation…it is really nice to have an organisation in contact with you that knows where you are at. Text message participant S12 I just liked the fact that it kind of told you what to expect … if someone kind of gives you what to expect, then you anticipate these changes, and you can look out for them. So I really liked that quite a bit … I think what I found most useful was… some of the changes to expect … when to introduce solids, I don’t know any of it … it was all useful to me. Text message participant S1 I just think if you guys need government funding to get this program into new mothers’ hands, I think it is so important … I would highly recommend other mothers to be a part of this program. It has been useful and It has been a really great way of adjusting to being a new mother. Text message participant S1 I would like every, at least first-time mum, to experience all this. I think it’s a blessing to have such a program. Telephone call participant T12 Along the way sent little gifts as well … one in particular that we were given was a little bowl and spoon. it’s her favourite one, we use it every day. Text message participant S4 I think a lot kind of focus on looking after myself as well as her. I think that was the message that I took … and then the exercise which I was a bit more focused to do. Telephone call participant T9 I think I wasn’t quite the right demographic as most women, because I already had a lot of health support and health information … for example, I am breastfeeding and my mum breastfed me until I was three … since birth, they haven’t been useful. I haven’t really learned anything new … it’s more just kind of confirmation of what I’ve been reading. Telephone call participant T10 I had all the help possible. The nurse I dealt with, was really helpful … she was not very judgmental … my suggestion about the program would be … if there is funding … it would be really good to actually meet the nurse. just meet the person once. Telephone call participant T13 At four or five months, I was really unwell … I was in hospital for six weeks … I could not take my baby with me, so I had to stop breastfeeding … I kept getting SMSs in the time period saying ‘don’t forget to keep breastfeeding … which actually made me miserable. Text message participant S7 “Whenever I am worried about something, I text Healthy Beginnings … and they answer me straight away so I am really happy with it … they answer me as best as they can do … I can ask questions and they reply quickly, so it was even better.” Text message participant S8
2. Mode of delivery	2.1 Telephone calls	It was nice that the nurses on the phone, they listened a lot. They didn’t bully you or push you or anything like that, which was really nice … it’s nice just to have someone who listens and doesn’t criticise. Telephone call participant T10 It was always very difficult coordinating the phone call time … That’s the story of your life. Maybe because I don’t have a routine baby. I wouldn’t know for sure when I was going to be free on any day, so often I would call when it was a good time for me, but of course the nurses would be busy. Often, we’d play phone tag for weeks trying to get hold. Telephone call participant T1 I feel like if you were someone who had less support than I do, need something before the baby comes … need something that follows up after those three months … yeah I think the telephone consultations would be very useful if everyone had access to them ideally. Telephone call participant T1 I also found it useful just to engage with somebody else … sometimes you become isolated. It’s good to have somebody else check in on you now and then…I could actually discuss topics that reflected my need. Telephone call participant T2 The phone calls have been beneficial … individualised feedback … They would give very practical advice and very easily … they would explain things until I understood them myself and not just as if it was to a whole group of people. Telephone call participant T3 It was good to talk to a nurse … if you had concerns about something … I got to ask them a question via text message and they came back to me the next day with a response … it was comforting to know it had come from a health professional rather me having to go down to the doctor and probably get the same answer. Telephone call participant T13 I think those phone calls are great and I think they should be recommended to all. Telephone call participant T6
	2.2 Text messages	I didn’t really benefit very much from the text messages. I think the pamphlets were the best … I did look at the text messages. That’s not to say that the text messages weren’t useful. I just found the booklets more useful. If you were to get rid of the text messages, and keep the booklets. You do it the other way. I would definitely keep the booklets. You didn’t need … I feel like it was a nice addition, but I don’t feel like it’s necessary to get the text messages. Text message participant S1 I would be thinking about something was going on with breastfeeding or something and the next day I would get, just by chance a message … it kind of made me go, yes I am doing the right thing … I think I was probably happy with the text messages because I often didn’t have time for a telephone call. But maybe a mix of the two … Because text messages you are not as likely to remember it all, so may be a phone call or something might have built that … I think that everyone is different so I think you need to give kind of a choice … probably recommend a couple of phone calls in there. Text message participant S5 When I receive the text messages, when you are home alone … at home with the baby, when you get a message like that, it sort of brightens your day … oh, someone is thinking about me … It does help and they are just little hints to keep you going … just remind you of things you may have forgotten … reminds me of information I already knew. Text message participant S3 I think even that, if your hardest time is in the evening, if it takes 24 h for someone to respond to your text messages anyway … It’s nice to have, but it’s not really that beneficial. Text message participant S1 “One of the text messages was incredibly de-motivational … It always sounded just very sort of automated, a marketing message, not like you could have a two-way conversation.” Text message participant S6 If you are still breastfeeding, make sure you go and have some rest or something like that … I know the intention was good but probably a bit patronizing … I think, probably in a text message you can’t fit enough information. So it would probably be a link or something like that … because I think there just wasn’t enough information. Text message participant S9
	2.3 HB Booklets	It was interesting to read the literature (booklets) … there would be differences between what the literature … and what the other literature was saying … for example, do you start solids at four months or six months? Telephone call participant T1 Well, my husband’s family were pushing very strongly that I feed him things like custard, sweetened with honey and things like that. I was able to say, ‘Look, the research says we can’t give them honey and we should avoid milk, and stuff like that.’ It was handy to have a source of authority to say that this is why I’m not feeding him those things. Telephone call participant T1 Sharing the booklets with my partner … here’s everything that we have been talking about lately, in one place. Telephone call participant T8 I have always put the booklet in front of my partner and asked if he read it and we talked about it … He has been the only person really that I have shared my experience with … Telephone call participant T9 I think what I found most useful was like I said, just some of the changes to expect at the certain times for your child, and kind of when to introduce solids. I didn’t know any of it. So, it was all useful to me … my husband, he also found it really useful, so we both kind of have the same views on the content. Text message participant S1 The booklets sort of explains what to start feeding them, when the time is right and … just new things that they are going to be learning at that stage … I have lot of kids in my family, my siblings and that … but the booklets did help with what is really right for them at this stage.” (Participant T10) “It’s been pretty good. It’s kind of covered at each point along the way … the main milestones, any questions I had about breast feeding and that sort of thing were answered. Telephone call participant T11 The booklets are really helpful … even if you have support, this booklet also comes in as additional support … I would say if anyone doesn’t understand what happens in the first three months, or three to six months how a baby should grow, I think the booklets supposed to clarify the doubt which is good. Telephone call participant T12 Easy to understand, it was really helpful … particularly the ones that have a little bit of information about the routines, feed and sleep … it was kind of nice how it arrived at the time that you needed it. Text message participant S4 Because it is over a period of time and it is small pieces of information that are relevant to the stage of your baby it is something that you can take in and refer back to, but without it being a huge amount of information at once … I have referred back to it a lot about food and things like that. Text message participant S5 If you were to get rid of the text messages, and keep the booklets. I would definitely keep the booklets … Text message participant S1 Probably the thing I’ve found most useful is the booklet that comes because they just give quite a good overview of what’s happening in those months … Probably didn’t find the text messages as helpful. Text message participant S2 I didn’t find the booklets as useful … It just wasn’t information I needed … The booklets just had a lot of information that wasn’t specifically relevant to me … They just weren’t relevant to me. I’m sure they’re helpful for other people … I found it useful just to engage with somebody else … Telephone call participant T2 The booklets, some of it was common sense, but nothing was ground-breaking or new, but it was just confirmation of what we already had read from other sources … a few of the dietary things were interesting for example, having no salt for the baby’s food … Telephone call participant T10 I’m a second time mother. I forget from the first one … The booklet is new information for me. May be the first one I do the wrong thing, you know … so this one is good for me … The booklet is useful … it’s easy and then. because my English is not very good … the booklet is easy, I can understand … I can see exactly what I want inside. Telephone call participant T5 I didn’t have enough time to sort of go online and read through but I did read through the booklets because it was there, in my hand. Telephone call participant T6 I’m usually someone that is not into paper … at least the booklet, I always put it next to my bed … it was there visible, so I could pick it up and read it and look at it … I found the booklets very useful. Text message participant S4 I have found the booklet quite helpful, I would sit down and read that whilst eating lunch or having a coffee or something. Text message participant S12 I can understand English, so it was easy. It was described in a very easy way, the booklet … in terms of the nurses calls may be sometimes a little bit of tension but it’s fine. Telephone call participant T7 I have a little folder of them … brochures that you sent. tip sheet from may be Tresilian or Karitane, that I referred to several times … some kind of department of health brochure about healthy eating and screen time.” (Participant T14) I did have a couple of books that had been recommended or people had given me … I did actually buy a book called Baby Love … I rarely even needed to look at it because of the information that was provided by you guys. Text message participant S4 I think they could have been delivered a bit earlier, because my baby came a few weeks early with my preeclampsia. It felt like by the time the booklet has arrived, I’d already researched that stage. Maybe if they’d come a month earlier it would have been more helpful. Telephone call participant T1 In a couple of instances there was some general information that I might have seen it somewhere else … it reminded me when it came … Control participant C1 The information in the booklets was fine but it wasn’t necessarily information that I couldn’t have found anywhere else … it’s very basic information that people are probably getting from other places. Control participant C4
3. Intervention contents	3.1 Breastfeeding	It’s been really helpful … I had some questions related to breastfeeding and weaning … I tried to go to Breastfeeding Australia but they’re very anti-weaning, which isn’t particularly helpful when you just need advice on how to do it properly … I found the nurse was able to provide me with really good, helpful advice that meant that weaning was a painless experience for both the baby and myself. Telephone call participant T2 Because of … whatever reasons, my milk stopped after one month … I was like sad that I couldn’t give the best to my son … the nurse’s call really helped me to overcome the stress … sometimes you can feel guilty if you are not giving best to your child. I think the nurses did give quality thoughts and how other mums may be suffering. Telephone call participant T12 I stopped breastfeeding … at five months old … I felt the booklet was encouraging me to continue to breastfeed her … but I felt a little bit of pressure … we had breastfeeding issues … so I kind of felt like, if I didn’t breastfeed her that I was being judged. Text message participant S1 I saw a lactation consultant and I also went to the breastfeeding drop-in clinic. I also went to a twins breastfeeding class at RPA. Text message participant S2 I was having trouble breastfeeding … when he was first born, so I actually had to physically go see a lactation consultant … I think telephone or text messages wouldn’t have been enough … physically going to the lactation consultant was I think more helpful. Control Participant C5
	3.2 Introduction to solids	I thought the dietary reminders and recommendations are helpful … best thing that I have gained from the program Telephone call participant T9 Information on ‘introduction to solids’ was useful because I have no idea what to give the baby when he can start solids … In terms of feeding solids may be some more information is better because … I am still feeding him by the spoon. So may be some more information on how to let him eat by himself. Telephone call participant T4 You guys had some extra … leaflets in the one that came, solids. That was really useful. I’m not sure, if that was from you or the local health centre, but they also sent a booklet that showed how to prepare the food. So, what consistency for what age. That was good. Text message participant S1 I got it from a range of places … solid information session at the clinic, baby book, internet, clinic…few trusted books. Telephone call participant T8 I introduced meals formally to her at six months … I took a class on starting solids with the early childhood centre and I was also given some information from my doctor…i also spoke to friends and things like that. Control participant C7 My GP was very strongly pushing (to start solids) from four months but I was worried that once he went to solids my baby wouldn’t want to breastfeed anymore. Telephone call participant T1 I have to say for that bit, I was a little bit confused because there were so much conflicting advice I was getting … I actually because I had so much milk, I did exclusively breastfeed her until six months and the started solids at six months … I felt like there was a lot of conflicting advice out there about when I should have started her on solids. Text message participant S4 Feeding him little taste of things at about four months … so he probably started eating solids at about five months … I did get it from Baby Love … I looked at both the Allergy Association and the Breastfeeding Association website … one says start at four months and the other one says start at six months … so I think I just kind of went somewhere in between. Control participant C1 Sometimes it was a little frustrating to get so many mixed messages from different professionals … I had my husband’s grandma giving me lots and lots of advice on what to do to help him feed … so you had to stick to your guns and in other things you had to be flexible. Telephone call participant T10 The nurse said don’t give solids to the baby at three months … The reason why nurse said not to bring in solids is because his digestive system was just growing … I just told them (family and friends) my husband doesn’t prefer it … because my husband spoke more with the nurse. It was easy for me to just follow my husband … My husband is more into my child’s pattern than me. He introduced solids only after eight months I think. Telephone call participant T12 I did ask the nurse and she just recommended to give him the solid food more and to reduce his milk … after she recommended and I think he is eating the solid food more and his poo is normal… I think that resolved the problem … I think this is better, it’s natural way. Telephone call participant T12 The only thing that really stood out was the breastfeeding fine until six months and start solids. I didn’t do that with my other children. I only done it with this one because I read it in the book. I done it a lot earlier. Text message participant S10 It wasn’t really going into great detail that kind of allows you to make up your own mind what vegetable you are going to feed … but it was just good for things like making sure that you can get the whole family to eat at the same time … sort of more general stuff. Text message participant S12 I am from Russia and we have a very different solid tradition … I think that in Australia it is much better for children, so I actually adopted the one that I found in your booklet … This is the thing that you probably should include in your booklet. I started solids with rice cereal as recommended … I probably made a mistake … I had a problem with poo … and then I read that it is not very good thing to start from rice cereal. Text message participant S13 I would have liked to have someone to discuss the food and stuff like that … I thought they would have guided in what food … what is good and what is energy and everything. Control Participant C2
	3.3 Tummy time	Sometimes mothers know a lot of things but when someone talks to them about tummy time … it’s kind of like a reminder … oh no, that’s right, we’ve got to do that today. Telephone call participant T6 The booklets reminded me about tummy time and I really got onto tummy time … I am sure it’s because I got a booklet and it told me what to do … I knew I should be doing tummy time but the booklets reminded me … the booklet is a good reminder. Telephone call participant T14 Tummy time, my son does it by himself. I think may be two months I tried to put him over the tummy but then he was on his tummy by himself most of the times. Telephone call participant T12 I don’t do it a lot actually, just because I was so busy. Text message participant S2 At every point from that health net news, from the community … mothers’ group … then I remember seeing it in the pamphlet … started at two weeks … Control participant C1
	3.4 Screen time	My baby watches TV … I know she shouldn’t … I need to do things like go to the toilet, or cook dinner … it’s just in the background … she doesn’t sit and watch it, she plays, dances and does different stuff. Text message participant S1 I thought that you shouldn’t really give them any screen time until they were two or something … I think I looked up recommendations. Control participant C1 A lot was stressed on tummy time and about screen time, and I’m glad I wasn’t doing screens anyway … I usually didn’t have any screen time with my daughter. Telephone call participant T6 If I need to go to the bathroom, the only way I can go to the bathroom … is by putting her in a chair in front of the TV … may be 15 min a couple of days a week when I am with her alone. Telephone call participant T8 I don’t give them any screen time except when they are going totally crazy. Text message participant S2 I don’t give my baby all the gadgets, no tablets, iPads but she, every kid is born with that, she does watch laptop, YouTube for about an hour a day, that’s all … in the entire day she watches around one hour. That’s fine, she is already 14 months. She is learning out of it. Control participant C3
	3.5 Sleep and settling	We ended up going to Karitane and getting some sleep help. Maybe the content was the same, but I think with the position of being really sleep deprived, and having a child that doesn’t settle, just what you’ve got in a booklet, you kind of need some hands-on help. Text message participant S1 The nurses used to tell me how many hours our baby should sleep and how to put the music on or try to give them the environment where he can sleep more without disturbing. Telephone call participant T12 Mine’s not really good at self-settling at all so we do a lot of co-sleeping … The booklets did help. I think they made me feel like it was normal. Text message participant S3 I definitely supplemented especially the sleep parts with some other books that I had … that was the one thing she struggled with unfortunately … Tresillian even came out to help us as well … I think I needed a bit more information on that. Text message participant S4 Regarding sleep pattern I actually didn’t follow because my young one … he needs to wake up to have some breastfeeding at night … To my knowledge many Chinese, we just follow the child’s sleeping pattern and didn’t force the child to do any changes for his sleeping. Text message participant S11
	3.5 Mother’s self-care	And how difficult it is to actually do anything with the baby, and how I need to have it planned, and all that sort of stuff … that probably would have been helpful but it also would have been, yes, have him call. Tell him. Control participant C1 The practicality of exercising, that didn’t happen … but what I did do is I changed my whole diet … It’s all about trial and error. I didn’t think there is anything wrong with their recommendations, it’s just that it wasn’t practical at the time to do something. Telephone call participant T6 To be honest, I don’t remember. I don’t know how many of them you might have sent, but I don’t remember … I think I definitely tried to do some exercise … Text message participant S4
	3.6 Goal setting	I liked how they had goals, I think that was good … I like how they summarised the call and sent you emails with your goals written down. Sometimes I would pass those emails on to my family just to let them know what stage I was up to … One of my goals for exercise was to get back into the dancing and I haven’t been able to reconcile it with my responsibilities as a mother. I just have to wait till the kids have grown up and then try it. Telephone call participant T1 One of the goals I had was to reduce the breastfeeding, and my nurse gave me strategies in order to reduce the breastfeeding, I was able to do that. Telephone call participant T2
4. Contextual/psychosocial issues	4.1 Demands on motherhood	I probably struggled initially to adjust to the life change. Maybe if there was more information at the beginning that was around … a lot of women feel the same way … adjusting to being a mother is really hard … It is really hard to be a mum. Text message participant S1 I guess the one thing that I probably struggled with was there’s a lot of expectations on mothers … Trying to balance expectations in terms of returning to work, having a healthy baby, giving them the right food. The advice that you get in terms of … it’s incessant. Telephone call participant T2 At four or five months, I was really unwell … I was in hospital for six weeks … I could not take my baby with me, so I had to stop breastfeeding … I kept getting SMSs in the time period saying ‘don’t forget to keep breastfeeding … ’which actually made me miserable. Text message participant S7
	4.2 Competing issues	In four to five weeks I’ll be going to work … so that is the major concern at the moment. That is one of my biggest challenges … separation anxiety as well. It’s like a big storm for me at the moment … that’s my biggest concern. Telephone call participant T6 I am back at work now. We had financial troubles when I was going through with my maternity leave, but none of it prevented me from the program. Telephone call participant T11 It just so happened that just after my daughter was born, my mum got cancer … a lot of support needed … being directed at thinking about her rather than looking after the baby … it was just bit of a hard slog for a few months trying to manage it all. Control participant C4
	4.3 Emerging health issues	Baby had late-onset group B Strep … She was in hospital for two weeks … she had really severe reflux, and I was trying to breastfeed her and I got mastitis twice. Telephone call participant T2 When your baby is not really adapting well to the guidelines, it is more how to deal with the deviation … what can I do instead … people’s experiences would be so varied … you wouldn’t be able to fit that all in. Telephone call participant T8 I found all of that kind of information (feeding and eating) frustrating for me personally because I wasn’t able to follow it due to swallowing difficulties … I’d feel like my kid was really delayed because he couldn’t do the things … in the literature or he couldn’t do the things other kids were doing. Telephone call participant T10
